# Evaluation of stability and safety of equine mesenchymal stem cells derived from amniotic fluid for clinical application

**DOI:** 10.3389/fvets.2024.1330009

**Published:** 2024-02-14

**Authors:** Eun Young Kim, Eun Ji Lee, Ryoung Eun Kim, Tae Young Kil, Min Kyu Kim

**Affiliations:** ^1^MK Biotech Co., Ltd., Daejeon, Republic of Korea; ^2^Division of Animal and Dairy Science, College of Agriculture and Life Science, Chungnam National University, Daejeon, Republic of Korea; ^3^Department of Social Welfare, Joongbu University, Chungcheongnam-do, Republic of Korea

**Keywords:** equine (horse), amniotic fluid, mesenchymal stem cells, long-term culture, stability and safety for cell therapy

## Abstract

Amniotic fluid mesenchymal stem cells (AF-MSCs), which can be obtained from fetal tissue, reportedly have self-renewal capacity and multi-lineage differentiation potential. The aim of this study was to identify the biological characteristics of AF-MSCs and evaluate their stability and safety in long-term culture. To confirm the biological characteristics of AF-MSCs, morphology, proliferation capacity, karyotype, differentiation capacity, gene expression level, and immunophenotype were analyzed after isolating AF-MSCs from equine amniotic fluid. AF-MSCs were differentiated into adipocytes, chondrocytes, and osteocytes. Immunophenotype analyses revealed expression levels of ≥95% and ≤ 2% of cells for a positive and negative marker, respectively. Analysis of the MSCs relative gene expression levels of AF-MSCs was approximately at least twice that of the control. The endotoxin level was measured to verify the safety of AF-MSCs and was found to be less than the standard value of 0.5 EU/ml. AF-MSCs were cultured for a long time without any evidence of abnormalities in morphology, proliferation ability, and karyotype. These results suggest that amniotic fluid is a competent source for acquiring equine MSCs and that it is valuable as a cell therapy due to its high stability.

## Introduction

The development of cell therapy products has progressed in various ways using stem cells for allogeneic treatment. Mesenchymal stem cells (MSCs) are less of an ethical concern than embryonic stem cells, have great plasticity, and secrete growth factors or cytokines to regulate the immune system of the host through paracrine effects (1, 2). Thus, MSCs are considered suitable for regenerative therapy.

As stem cells are used directly in living organisms for cell therapy, the safety of these cells must be guaranteed. Cell proliferation occurs through mitosis, forming two daughter cells that are identical to the parent cell. During repeated mitosis, cells sometimes fail to maintain a stable and safe state, which may alter gene expression, arrest cell division, or drive the formation of cancer cells (3, 4). For the uniformity and efficiency of cell therapy, it is necessary to stably culture stem cells. This study aimed to evaluate the stability and safety of stem cell cultures for treatment.

Adult stem cells can differentiate into various cell types, including adipocytes, chondrocytes, and osteocytes (5). The therapeutic application of MSCs, a subtype of adult stem cells, is a promising treatment for tissue regeneration and repair (6). Adult MSCs can be obtained from whole-body tissues, such as bone marrow (7), adipose tissue (8), Wharton's jelly of the umbilical cord (9), umbilical cord blood (10), peripheral blood (11), synovial membrane (12), adipose tissues (13), placenta (14), amniotic membrane (15), and amniotic fluid (16). Amniotic fluid (AF) is a promising source of stem cells because it contains various cell types derived from the developing fetus (17, 18). AF-derived MSCs (AF-MSCs) have several advantages, including a reduced risk of immunological rejection and oncogenesis, pluripotency suggested by their origin from the proximity of the embryonic inner cell mass, and a less invasive collection procedure (19). AF-MSCs are used in regenerative therapy as the cells can be used to obtain tissues in large quantities and have a low risk of immune rejection (20, 21).

AF-MSCs have been widely used as stem cell treatments. However, their stability and safety are unclear and must be confirmed. The aims of this study were to identify the characteristics of equine AF-MSCs and evaluate the stability and safety of these cells for the development of equine AF-MSCs-based therapies. The characteristics of AF-MSCs were confirmed through morphological evaluation, determination of differentiation capacity, and marker expression analysis. After characterization, karyotyping was performed during prolonged culture of AF-MSCs to evaluate their stability by investigating the presence of mutations that may occur during long-term cell culture (22). Measurements of endotoxins and mycoplasma were performed to inspect cytotoxicity, as the absence of cytotoxicity must be guaranteed before using stem cells for therapeutic purposes (2).

## Materials and methods

### AF-MSCs isolation and culture

The study protocol complied with the conditions of The Guide for the Care and Use of Laboratory Animals and was approved by the Ethical Committee of Chungnam National University (Approval No. 202203-CNU-002). The AF samples used in this study were obtained from eight mature mares at the Songarm Horse Breeding Farm in South Korea. AF was obtained through normal delivery without invasive surgery. Each AF sample was collected from the amniotic sac using a 50 ml syringe equipped with an 18-gauge needle immediately before and after birth. The sample was diluted in phosphate-buffered saline (PBS, Gibco, Carlsbad, CA, USA) containing 1% penicillin-streptomycin (P/S, Gibco) and centrifuged at 1,500 x g for 7 min. After isolation, the prepared AF was washed three times with PBS and seeded on a cell culture dish coated with 0.1% gelatin (Sigma-Aldrich, St. Louis, MO, USA). Cultures were grown in Roswell Park Memorial Institute (RPMI) 1640 medium (Gibco) containing 15% fetal bovine serum (FBS, Gibco) and 1% P/S at 38.5°C in an atmosphere of 5% CO_2_. For culture expansion, cells obtained from AF were maintained in Dulbecco's modified Eagle's medium (DMEM, Gibco) supplemented with 15% FBS and 1% P/S at 38.5°C in 5% CO_2_. Finally, according to the cell establishment results (Supplementary Table 1), lines 3 and 4 amniotic fluid cell lines were used in this study.

### MSC characterization

The induction of mesenchymal tri-lineage differentiation was performed using the StemPro Differentiation Kit (Gibco). The cells were stained after 7, 14, and 21 days of adipogenic, chondrogenic, and osteogenic induction, respectively. Differentiation into adipocytes, chondrocytes, and osteocytes was demonstrated by staining with Oil red O, Alcian blue, and Alizarin red S solution (all from Sigma-Aldrich), respectively. Cells that were not induced were also stained with each staining solution. Cells were observed by microscopy using an Eclipse TE2000-U microscope (Nikon, Tokyo, Japan).

Cells were harvested when 70–80% confluent with 0.05% trypsin-EDTA (Gibco) and subjected to mRNA extraction and real-time polymerase chain reaction (PCR). mRNA was extracted using an RNA extraction mini kit (Qiagen, Hilden, Germany). After RNA purity was determined using a nanodrop spectrophotometer (BioSpec, Bartlesville, OK, USA), cDNA was synthesized from 1 μg total RNA using a commercial cDNA synthesis kit (Bioneer, Daejeon, South Korea). Real-time PCR was performed with equine primers based on the National Center for Biotechnology Information Equus caballus sequences available using a SYBR kit (Bioneer); the primers used are listed in Table 1. The mRNA levels of β-actin; pluripotency markers (Pou5f1, c-Myc, and Klf4); and MSCs markers (Pax6, endoglin, integrin β1, and HCAM) were analyzed. PCR amplification was performed at 95°C for 4 min, followed by 40 cycles of denaturation at 95°C for 30 s, annealing at 58°C for 30 s, and a final extension at 73°C for 15 s. Data were normalized to β-actin RNA levels. The relative fold gene expression of the sample was calculated using the delta-delta Ct method, and the amount of gene expression of the AF-MSCs compared to that of horse skin cells was calculated using the following equation:


$$\begin{array}{l} \Delta \Delta \text{Ct }=\;\Delta \text{Ct }\left(\text{AF}-\text{MSCs}\right)-\;\Delta \text{Ct }\left(\text{horse skin cell}\right)\; \end{array}$$

**Table 1:** Primer list.

**Name**	**Accession No**.	**Primer sequence**	**Base pair**
beta-Actin	NM_001081838.1	(F) GGATGCAGAAGGAGATCACAG	125
		(R) CTGGAAGGTGGACAATGAGG	
POU5F1	XM_00149010	(F) TCTCCCATGCACTCAAACTG	197
		(R) AACTTCACCTTCCCTCCAAC	
c-Myc	XM_023655154	(F) GCCCATAAAATTGCCAAGAGG	132
		(R) AGCCCTGACCTTTGAATGAC	
Klf4	XM_023629843.1	(F) ACCTCGCCTTACACATGAAG	218
		(R) TGGTTTCCTCATTGTCTCCTG	
PAX6	XM_023646553.1	(F) TGTTTGCCCGAGAAAGACTAG	224
		(R) AGAGGTGAAGGATGAAACAGG	
Integrin-ß1	XM_005606848.3	(F) CTTATTGGCCTTGCATTGCT	169
		(R) TTCCCTCGTACTTCGGATTG	
HCAM	XM_005598012	(F) ATCCTCACGTCCAACACCTC	165
		(R) CTCGCCTTTCTTGGTGTAGC	
Endoglin	XM_003364144	(F) AAGAGCTCATCTCGAGTCTG	162
		(R) TGACGACCACCTCATTACTG	

Flow cytometry was performed to confirm the expression of marker antigens recommended for MSC definition using a FACS Canto apparatus (BD Biosciences, New Jersey, USA). Cells were fixed with 4% paraformaldehyde (Thermo Fisher Scientific, MA, USA), and stained with antibodies against CD29 (phycoerythrin [PE]-labeled anti-human CD29 antibody; BioLegend, San Diego, CA, USA), CD44 (PE-labeled anti-mouse/human CD44 antibody; BioLegend), CD90 (PE-labeled mouse anti-rat CD90/mouse CD90.1; BD Biosciences), CD105 (fluorescein isothiocyanate [FITC]-labeled mouse anti-human CD105; Bio-Rad, Hercules, CA, USA), CD14 (porcine/equine CD14 antibody, R&D Systems, Minneapolis, MM, USA), CD34 (FITC-labeled mouse anti-human CD34; BD Pharmigen, Franklin Lakes, NJ, USA), CD45 (FITC-labeled mouse anti-human CD45; Southern Biotech, Birmingham, AL, USA), and major histocompatibility class II (FITC-labeled MHC class II antibody, clone CVS20; LSBio, Seattle, WA, USA). Fluorescence-activated cell sorting (FACS) histogram data were analyzed using the FlowJo software (FlowJo, Ashland, OR, USA).

### Colony-forming unit assay

Cells were seeded at a density of 20 cells/cm^2^ and cultured at 38.5°C in an atmosphere of 5% CO_2_ for 10 days. Colonies were stained with the Diff-Quick kit (Sysmex, Kobe, Japan), washed three times with PBS, and counted.

### Doubling time analysis

To evaluate cell proliferation, the doubling time (DT) of each passage was investigated every 48 h. DT was calculated when the number of cells was twice the number of initial cells. Mean DT was calculated using the following formula:


$$\begin{array}{l} \text{CD}=\text{log}\frac{\frac{\text{Ni}}{\text{Nf}}}{\text{log2}},\text{DT}=\frac{\text{CD}}{\text{CT}} \end{array}$$

where CD represents the cell duplication factor, Ni is the number of seeded cells, Nf is the number of cells at confluence, and CT is the culture time. Data from four independent experiments are reported. The average DT of each group was calculated by dividing the passage number by 10 from passage 0 to 50.

### Karyotype analysis

Cells at passages 10, 20, 30, 40, and 50 were collected for karyotyping. The cells were cultured for chromosome preparation. In brief, AF-MSCs were grown in a 25 cm^2^ culture flask with DMEM medium supplemented with 15% FBS and 1% P/S at 38.5°C in a humidified incubator containing 5% CO2. Before harvesting, 0.2 μg/ml of colcemid (Gibco) was added for 1 h to induce metaphase chromosomes. Subsequently, the cells were incubated with a hypotonic solution containing 0.06 M KCl (Sigma-Aldrich) for 15 min and fixed in Carnoy′s solution (3 parts of methanol and 1 part of acetic acid) for 30 min. Following the above fixation procedure, repeated three times, and centrifugation for 10 min at 150×g, the AF-MSCs were dropped onto glass slides for the specimen of GTG -banding. G-banding was performed following the procedures outlined in the method by Sohn et al. (23). The slides were immersed in 0.1% trypsin for 15 s and washed in cold Dulbecco's PBS without calcium and magnesium (DPBS, Gibco). After washing, the slides were stained with 0.04% Leishman′s solution (Sigma-Aldrich) for 5 min and examined by light microscopy.

### Microbial contamination assay

Mycoplasma contamination was assessed using the Myco-Sniff-Valid Mycoplasma PCR detection kit (MP Biomedicals, Irvine, CA, USA). Mycoplasma detection sample preparation was performed using the cell boiling method. At least 5 × 10^∧^4 cells washed with PBS were heated at 95°C for 10 min, and the supernatant was utilized as a template in the PCR reaction. The PCR was executed at 94°C for 1 min, followed by 40 cycles of denaturation at 94°C for 30 s, annealing at 60°C for 20 s, extension at 72°C for 1 min, and a final extension at 72°C for 5 min. The evaluation data were validated with the internal control (170 bp) included in the PCR premix, and the results were interpreted through comparison with the mycoplasma positive control (260–280 bp).

### Endotoxin analysis

Endotoxin levels were quantified by measuring the amount of chromogenic substances derived from chromogenic substrates through the limulus amoebocyte lysate (LAL) reaction, ensuring the quality control of cell therapy products. The endotoxin testing employed U.S. Food and Drug Administration (FDA)-certified cartridges (Charles River Laboratories, Wilmington, MA, USA), meeting bacterial endotoxins test (BET) standards for photometric techniques as per the International Pharmacopeia. Concentrations were assessed in the conditioned medium in which AF-MSCs were cultured for 48 hours, following the protocol, using an Endosafe^®^ nexgen-PTS™ spectrophotometer (Charles River Laboratories). The test's validity was confirmed by examining the difference in reaction time between two repetitions of the sample (Sample CV), the difference in reaction time between two repetitions of the positive control (Spike CV), and the spike recovery rate.

### *In vitro* cytotoxicity test

Cell viability was assessed using the methyl thiazoyltetrazolium (MTT) cell proliferation assay kit (Invitrogen, Carlsbad, CA, USA) following the manufacturer's protocol. Briefly, cells were seeded in culture medium at a density of ~25,000 cells/cm^2^ in 96-well plates and incubated until reaching optimal population density. For the cytotoxicity positive control, 100% Dimethyl sulfoxide (DMSO, Sigma-Aldrich) was used. After incubation, the medium was removed, and 100 μL of fresh culture medium, along with 10 μL of the 12 mM MTT stock solution, was added to each well, followed by incubation at 37°C for 4 h. The cytotoxicity positive control group was analyzed after treatment with 100% dimethyl sulfoxide for 1 hour. For the negative control, 10 μL of the MTT stock solution was added to 100 μL of medium alone. After incubation, 100 μL of 0.01 M hydrochloric acid (HCl, Sigma-Aldrich) was added to a tube containing 1 mg of Sodium dodecyl sulfate (SDS) solution (Sigma-Aldrich) for each well, mixed thoroughly using a pipette, and the microplate was incubated again at 37°C for 4 hours in a humidified chamber. Data were then read in absorbance at 570 nm. Cell viability was calculated using the formula:


$$\begin{array}{l} \text{Cell viability }\left(\%\right)={\frac{\text{OD}\left(\text{570}-\text{650}\right)\text{e}}{\text{OD}\left(\text{570}-\text{650}\right)\text{b}}}^{*}\text{100} \end{array}$$

where optical density (OD) represents the measure of absorbance. OD (570–650)e is the value obtained by subtracting the 650 nm absorbance from the 570 nm absorbance of the test substance-treated well, and OD (570–650)b is the value of the negative control treated with the blank test solution. Data were reported from three independent experimental replicates.

### Statistical analysis

Statistical analysis was conducted using the GraphPad Prism program (GraphPad, La Jolla, CA, USA). For each experiment, a minimum of three replicates were utilized. Unpaired *t*-tests were employed for the analysis of CFU-F and real-time PCR data. Statistical significance was indicated at *P* < 0.05, *P* < 0.01, or *P* < 0.001, depending on the specific experiment.

## Results

### Isolation the AF-MSCs from equine amniotic fluid

**Figure 1:**
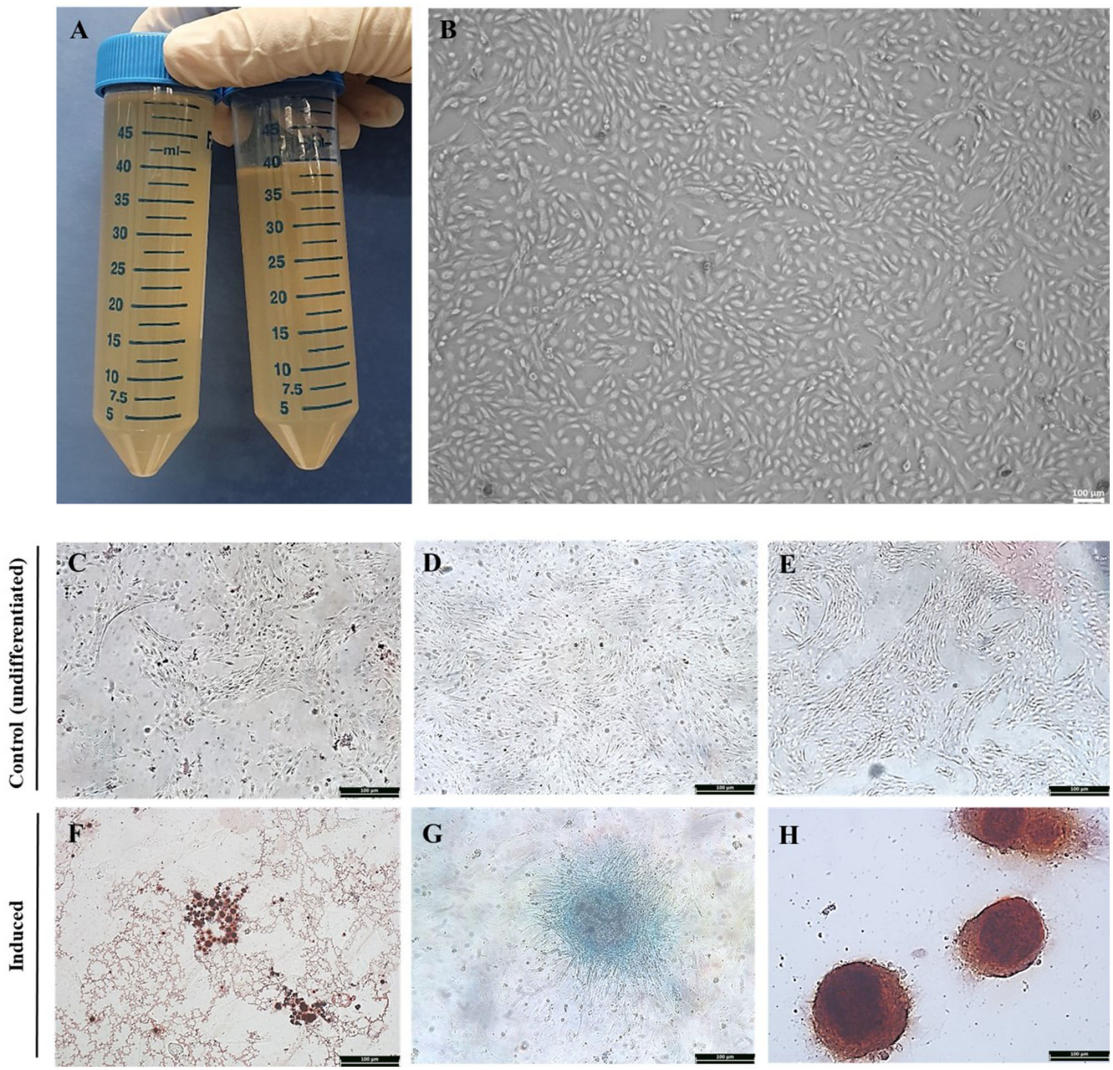
Isolation of equine amniotic fluid-derived stem cells and confirmation of differentiation ability. Equine amniotic fluid collection **(A)**. Morphology of cell isolated from equine amniotic fluid at passage 7. The MSC presenting a fibroblast-like characteristics and adherence to the plastic. The AFMSC was demonstrated under optical microscope in x100 **(B)**. The differentiation of AF-MSC into tri-lineages at passage 4. The adipogenic, chondrogenic, and osteogenic differentiation was confirmed by oil red O, alcian blue, and alizarin red S, respectively. The differentiation uninduced AF-MSC was not stained with oil red O [x100, **(C)**], alcian blue [x100, **(D)**], alizarin red S [x100, **(E)**], and induced AF-MSC was stained with oil red O representing oil droplets [x100, **(F)**], alcian blue representing connective tissue and cartilage matrix [x100, **(G)**], alizarin red S representing matrix calcium formation [x100, **(H)**].

Equine AF was collected by syringe aspiration in a 50 ml collection tube from the equine amnion following delivery of offspring (Figure 1A). Cultures and subcultures were established after the adherent cells were detected. Cell attachment was observed approximately 72 h after isolation from the AF. The morphology of AF-MSCs was observed at passage 7 using bright field optical microscopy. When observed in the absence of Diff-Quik (Sysmex) staining, the AF-MSCs showed a fibroblast-like form, adhered to a plastic tissue culture dish (Figure 1B).

### Tri-lineage differentiation of AF-MSCs

AF-MSCs differentiation was evaluated at passage four. AF-MSCs were induced to differentiate into adipocytes and stained positively with Oil red O (Figure 1F), indicating the presence of oil droplets in cells. After chondrogenic induction, AF-MSCs were positively stained with Alcian blue (Figure 1G), highlighting connective tissue and cartilage matrix. AF-MSCs were positively stained with Alizarin red S following osteogenic induction, revealing matrix calcium formation (Figure 1H). This stain detects matrix calcium formation. Undifferentiated AF-MSCs were not stained with any of these stains (Figures 1C–E).

### Marker expression in AF-MSCs

**Figure 2:**
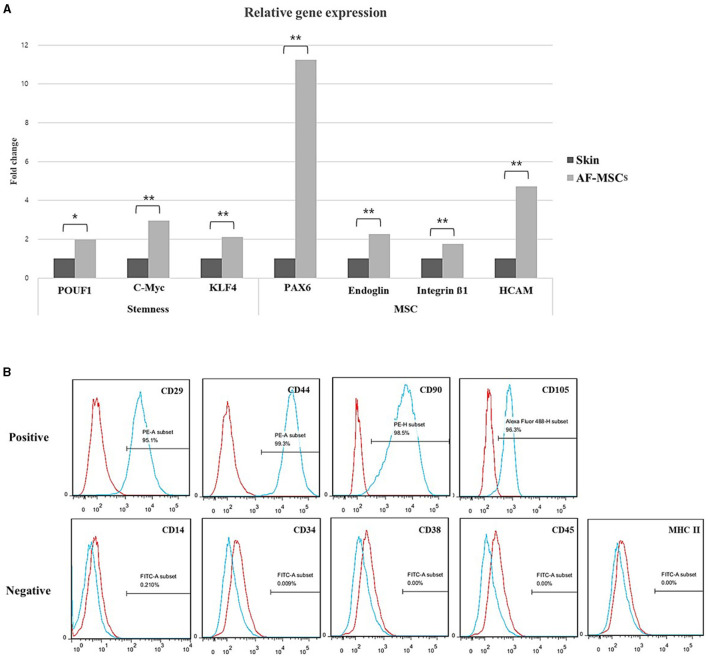
Stem cell marker variation. Relative gene expression level was normalized by expression level of target gene of skin fibroblast **(A)**. The graph and error bar represent mean of ΔCT value ± SEM. Significant difference represented ^*^(*p* = 0.0016) and ^**^(*p* < 0.001). Cell surface marker analysis of AF-MSC at passage 5 to 7 by FACS **(B)**. The AF-MSC were positive for CD29, CD44, CD90, and CD105, and negative for CD14, CD34, CD38, CD45, and MHC class II. Expression of IgG was used for negative control.

The marker expression in AF-MSCs was compared to that in horse skin cells; the relative levels are shown in Figure 2A. Pluripotency markers (POU5F1, c-MYC, and Klf4) and MSCs markers (Pax6, endoglin, integrin ß1, and HCAM) demonstrated significantly higher expression levels in AF-MSCs than in horse skin cells. Cell surface markers were analyzed using flow cytometry. As suggested by the protocols of the International Society for Cell & Gene Therapy (ISCT), the expression patterns of equine MSCs-positive markers (CD29, CD44, CD90, and CD105) and equine MSCs-negative markers (CD14, CD34, CD38, CD45, and MHC class II) were analyzed. All the MSCs-positive markers were expressed in >95% of the cells. The MSCs-negative markers were expressed in <2% of the cells (Figure 2B).

### Stable proliferation of AF-MSCs

**Figure 3:**
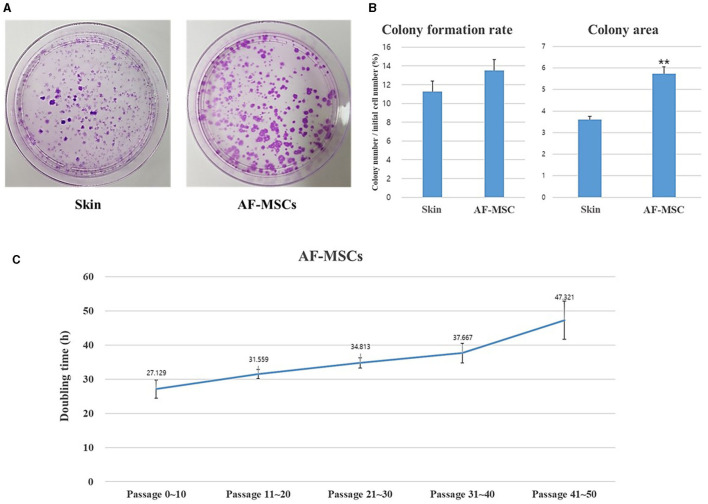
Analysis to evaluate the cell proliferation ability of AF-MSCs. Colony formation rate was evaluated at passage 5 during 14 days expanded in 100 mm cell culture dish **(A)**. The colony formation rate was calculated through the ratio of the number of colony to the number of cells initial seeded **(B)**. Significant difference represented **p < 0.001. The graph and error bar represent mean ± SEM. For the doubling time test of AF-MSC **(C)**, cells were seeded at a density of 200,000 cells per well of 6 well plates. The cell number was counted every 48 h. The graph and error bar represent mean ± standard error of the mean (SEM).

A CFU-F assay was performed to evaluate the self-renewal ability of AF-MSCs compared to that of horse ear skin cells (Figure 3A). Horse ear skin cell is a type of fibroblast; it is a skin-derived somatic cell and not a stem cell. Both horse ear skin cells and AF-MSCs formed hundreds of colonies in cell culture dishes. To evaluate the proliferative ability of the CFU-F assay, AF-MSCs and horse ear skin cells were seeded at a concentration of 20 cells/cm^2^, and the proportion of cells formed was calculated by counting the number of colonies divided by the number of cells initially seeded. Colonies were counted in four replicates; no significant difference was evident according to the *t*-test (*P* = 0.0669). The average percentages of colony formation of AF-MSCs and horse ear skin cells were 13.52 ± 0.80% and 11.25 ± 0.23%, respectively (Figure 3B).

Growth was assessed by measuring DT. AF-MSCs expanded and were sub-cultured to passage 50 (Figure 3C). DT of AF-MSCs from low to high passages showed a gradual increase when measured every 48 h (27.13±2.64, 31.56±1.34, 34.81±1.48, 37.67±2.86, and 47.32±5.56).

### Evaluation of cell stability and safety

**Figure 4:**
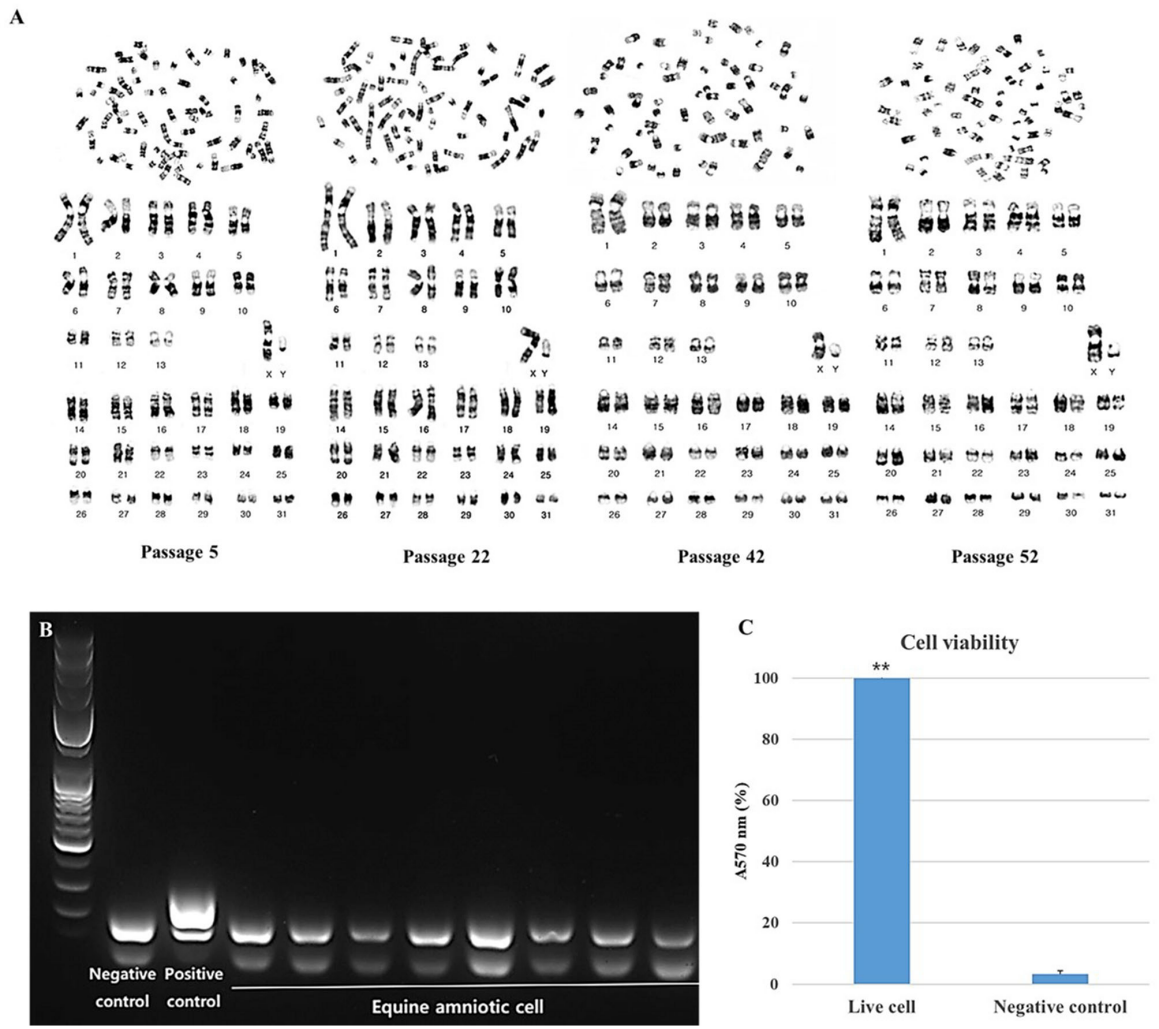
Safety evaluation analysis of AF-MSCs. Karyotype of AF-MSCs at passage 5, 22, 42, 52 showing 31 pairs of autosome and 1 pair of sex chromosome, a normal karyotype of horse **(A)**. Mycoplasma contamination test by PCR **(B)**. When mycoplasma is contaminated, bands appear in 264 to 277 base pairs. A band of the internal control appears in the 170 base pair as a negative control. In AF-MSC, bands appeared only at the internal control position. This indicates that AF-MSC is mycoplasma negative. The band that appears <100 base pairs is primer dimer. Bar graph representation of cell viability as determined by MTT assay **(C)**. Cell viability of AF-MSCs in the “Live cell” and “Negative control” treated with 3% DMSO was represented as the percentage of absorbance at 570 nm. The statistically significant difference between the two groups, denoted by the double asterisks (**p < 0.01).

The stability and safety of AF-MSCs were analyzed for clinical application in the field of stem cell therapy. Chromosomal mutations were confirmed through the equine karyotypes at various passages of AF-MSCs (Figure 4A). Horses have 32 pairs of chromosomes, with 31 pairs of autosomes and a pair of sex chromosomes. No chromosomal mutations were detected in AF-MSCs cells even after 50 passages. Microbial contamination was conducted to confirm the safety of the cell therapy, and the AF-MSCs were negative for mycoplasma detection (Figure 4B). Endotoxin levels in injectable products are the most important quality control test required by the FDA for all pharmaceutical products. The AF-MSCs were all detected at <0.5 EU/ml and so fulfilled the FDA criterion for therapeutic safety (Table 2). In cell viability analysis using MTT analysis, the average survival rate of the cytotoxic positive control group treated with DMSO was 3%, and AF-MSCS was 100%, confirming that there was no cytotoxicity (Figure 4C).

**Table 2:** Endotoxin concentration of AF-MSCs.

**Sample**	**Endotoxin concentration (EU/ml)**
EAF 1	<0.5
EAF 2	<0.5
EAF 3	<0.5
EAF 4	<0.5

## Discussion

The use of MSCs for horse musculoskeletal disorders has been extensively studied in recent years (24). MSCs can be obtained from various tissues in the body. Among these, fetal tissues such as AF, amniotic membrane, and the umbilical cord are plentiful and safe sources of MSCs (25, 26). These can be obtained easily and sufficiently without the need for surgery and the ethical concerns that can hinder the use of embryonic stem cells (15). Umbilical cord MSCs, in particular, reportedly have a lower proliferation capacity than AF-MSCs (25). Therefore, we explored MSCs from horse AF and successfully isolated them, establishing their cell lines and identifying their biological and therapeutic properties.

Cell proliferation was evaluated by measuring colony formation and DT. Another study reported that fibroblasts have superior proliferative capacity than typical MSCs and rapidly expand in culture dishes (27). In the present study, the colony-forming ability of AF-MSCs was not significantly different from that of fibroblasts, suggesting that AF-MSCs have remarkable proliferative capacity. The DT of AF-MSCs ranged from 27 to 48 h, depending on the number of passages. The proliferation rates of AF-MSCs were much faster than those of adipose-derived MSCs isolated from equine adipose tissue in another study (8). The DT of bovine amniotic MSCs exceeded 48 h in passage 10 (28), in contrast to the presently observed DT of <48 h after 40 or more passages. This finding suggests that differences in proliferation kinetics among different species or tissue origins are possible, which can be due to differences in culture medium components and culture conditions (29).

The stability and safety of cells must be proven before the cells can be directly transplanted in therapeutic procedures. In addition to general analysis, such as phenotype or gene analysis of cells applied for treatment, genetic characteristics through karyotyping for clinical applications can also be considered (30). Alterations in MSCs during long-term culture have been demonstrated previously. This includes malignant transformation and altered karyotype. MSCs with abnormal karyotypes displayed alterations in their morphology and phenotype, as well as functional aspects, including differentiation and proliferation. MSCs with abnormal karyotypes are highly oncogenic and a biohazard when injected *in vivo* (31). Therefore, it is important to ascertain that a normal karyotype is maintained to establish MSCs for cell therapy. In the present study, AF-MSCs did not show an abnormal karyotype, even after long-term culture, indicating that they expanded stably. Additionally, endotoxin and mycoplasma contamination must be ruled out prior to clinical application, as these can cause lethal shock in the living body (32). The endotoxin concentration and mycoplasma contamination results of AF-MSCs indicated that these cells were sterile.

The differentiation capacity is one of the minimal criteria for multipotent stem cells. Adipogenic, chondrogenic, and osteogenic differentiation can be demonstrated by staining with Oil red O, Alcian Blue, and Alizarin red S, respectively (33). Our results indicated that AF-MSCs have tri-lineage differentiation capability, showing positive responses to each staining solution, similar to other studies (34, 35). These differentiation characteristics are important for the therapeutic application of MSCs in musculoskeletal disorders, including osteoarthritis, which is a common disease in horses (36).

Gene expression analysis confirmed the therapeutic potential of AF-MSCs, based on their stem cell characteristics. The expression levels of the relevant genes were significantly higher in AF-MSCs than in skin fibroblasts. Pou5f1, c-MYC, and Klf4 are transcription factors that maintain pluripotency in embryonic stem cells and early embryos (37, 38). The high expression levels of the pluripotency and MSC markers Pax6, endoglin, integrin β1, and HCAM that we observed were consistent with those reported in previous studies (19, 39). The expression levels of Pax6 were particularly high. Pax6 has been reported in other studies as a gene expressed in MSCs with regenerative therapeutic abilities, especially eye or nerve regeneration abilities (40). Another study reported that AF induces enhanced retinal precursor cell generation from human retinal pigment epithelial cells (41). This was presumably related to the significantly high expression levels of Pax6 in our study.

Based on the minimal criteria for MSCs suggested by the ISCT, we examined whether AF-MSCs tended to be positive for CD29, CD44, CD90, and CD105 and negative for CD14, CD34, CD38, CD45, and MHC class II. In previous studies, equine MSCs highly expressed CD29 and CD44 (42). CD90 and CD105 expression levels differed according to their tissue origin (43, 44), whereas CD34, CD45, CD14, and MHC class II were not expressed (9, 36). In particular, MHC class II is an immunogenic antigen and a marker that must not be expressed to prevent immune rejection unless autologous tissue-derived cells are transplanted. Additionally, we further investigated CD38, a marker of inflammation-induced differentiation of monocytes (45). Although it is not a typical MSC-negative marker, CD38 was not expressed in AF-MSCs when the expression pattern in these cells was examined to improve the purity of MSCs.

## Conclusion

In conclusion, AF-MSCs exhibit the essential characteristics of MSCs outlined by the ISCT, demonstrating robust proliferative capacity, appropriate marker expression, and a notable differentiation potential. The stability and safety of AF-MSCs remain consistent even with prolonged culture and increased passages. Therefore, amniotic fluid emerges as a well-suited source for obtaining MSCs, positioning AF-MSCs as viable candidates for MSC-based therapeutic applications.

## Data availability statement

The raw data supporting the conclusions of this article will be made available by the authors, without undue reservation.

## Ethics statement

The animal study was approved by the Guide for the Care and Use of Laboratory Animals and was approved by the Ethical Committee of Chungnam National University (Approval No. 202203-CNU-002). The study was conducted in accordance with the local legislation and institutional requirements.

## Author contributions

EK: Conceptualization, Data curation, Investigation, Project administration, Writing—review & editing, Writing—original draft. EL: Formal analysis, Investigation, Writing—original draft. RK: Formal analysis, Investigation, Writing—review & editing. TK: Investigation, Resources, Writing—review & editing. MK: Conceptualization, Project administration, Supervision, Validation, Writing—review & editing.
