# Supplementary material for: Evaluation of stability and safety of equine mesenchymal stem cells derived from amniotic fluid for clinical application
Source: Front Vet Sci. 2024 Feb 14;11:1330009. doi: 10.3389/fvets.2024.1330009 (PMC10899390; doi:10.3389/fvets.2024.1330009)
Supplement: Supplementary file 1 [file Table_1.DOCX]

Supplemental Table. Amniotic fluid sample used for stem-cell derivation

| **Experimental**  **number** | **Cell establishment** | **Cell number** | |
| --- | --- | --- | --- |
| 1 | X | - |  |
| 2 | O | 22,000,000 cells |  |
| 3 | O | 438,000,000 cells |  |
| 4 | O | 99,000,000 cells |  |
| 5 | X | - |  |
| 6 | O | 16,000,000 cells |  |
| 7 | O | 3,000,000 cells |  |
| 8 | O | 27,000,000 cells |  |
